# Recent Insights into the Creation of Histone Deacetylase Inhibitors for the Treatment of Human Diseases

**DOI:** 10.3390/ijms26178629

**Published:** 2025-09-04

**Authors:** Pavel Yudaev, Yulia Aleksandrova, Margarita Neganova

**Affiliations:** Nesmeyanov Institute of Organoelement Compounds, Russian Academy of Sciences, Vavilova St., 28, Bld. 1, Moscow 119991, Russia; yulia.aleks.97@mail.ru

**Keywords:** cancer, Alzheimer’s disease, Parkinson’s disease, heart failure, psoriasis, histone deacetylase, histone deacetylase inhibitor

## Abstract

This review examines publications over the past two years devoted to histone deacetylase inhibitors for the treatment of cancer, diseases of the nervous, cardiovascular, digestive, and respiratory systems, and autoimmune diseases. The review covers various classes of histone deacetylase inhibitors depending on the zinc-binding group, in particular hydroxamic acids, benzamides, hydrazides, carboxylic acids, and cyclic peptides. The review pays special attention to the mechanisms of development of pathologies involving various isoforms of histone deacetylases. The review shows that, for the treatment of cancer, nervous, cardiovascular, respiratory systems, and autoimmune diseases, the most promising compounds are hydroxamic acids, and for the treatment of diseases of the digestive system, they are hydrazides and cyclic peptides. Variation in the linker and cap group of hydroxamic acids will allow the creation of an inhibitor selective for a specific histone deacetylase isoform. The review may be useful for molecular biologists, medical workers, and pharmacologists involved in the design of new drugs.

## 1. Introduction

**Figure 1:**
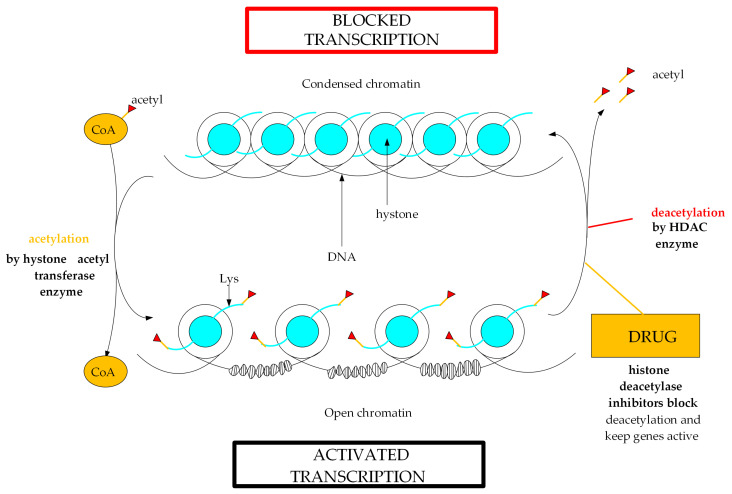
Brief diagram of histone acetylation and deacetylation in chromatin.

Histone deacetylase (HDACs) is a large family of enzymes that catalyze the removal of the acetyl functional group CH_3_C(O)O (acetyl) from the ε-AcNH lysine residue on both histone and non-histone proteins of various cellular localizations (Figure 1) [1].

Histone acetylation is required to maintain an open chromatin structure, which is associated with transcriptional activation [2]. Acetylation and deacetylation of histone lysine residues are in equilibrium, regulated by histone acetyltransferases in the presence of endogenous acetyl-CoA as an acetyl group donor and HDACs, respectively [3] (Figure 1). Dysregulation of HDACs disrupts the acetylation–deacetylation balance and contributes to the development of various pathologies [4]. The occurrence of pathologies is associated with the fact that deacetylation, catalyzed by HDACs, which prevails over acetylation, promotes an increase in the positive charge of the lysine amino group on the histone tail and high-affinity binding to the negatively charged phosphate groups of the DNA backbone, which condenses the chromatin structure, makes chromatin dense and inactive, and prevents genetic transcription [5].

In mammals, HDACs are classified into four distinct classes based on their structure, subcellular localization, and cofactor requirements: class I RPD3-like proteins (HDAC1, HDAC2, HDAC3, and HDAC8); class IIa (HDAC4, HDAC5, HDAC7, HDAC9) and IIb (HDAC6, HDAC10) HDA1-like proteins; class III SIR2-like proteins (SIRT 1–7); and class IV, which currently includes only the HDAC11 isoform [6].

HDAC1, HDAC2, and HDAC11 are localized predominantly in the nucleus, HDAC3, HDAC4, HDAC5, HDAC7, HDAC8, and HDAC9 are localized both in the nucleus and in the cytoplasm, and HDAC6 and HDAC10 are localized predominantly in the cytoplasm of the cell [7].

It should be noted that class IIa HDACs (HDAC4, HDAC5, HDAC7, HDAC9) do not have their own deacetylase activity and are pseudodeacetylases, since in their active center the catalytic tyrosine (Tyr976), conserved in all HDACs, is replaced by a histidine residue (His976). Class IIa HDACs act as scaffolds to attract catalytically active HDACs [8].

Class I, IIb, and IV HDAC enzymes hydrolyze the amide bond in the *ε*-N-acetyl-lysine residues of protein substrates by coordinating the oxygen atom of the acetyl group with a zinc ion Zn^2+^. Class III enzymes, unlike the other classes, which are zinc-dependent, do not have a catalytic site for zinc ions Zn^2+^, require nicotinamide adenine nucleotide (NAD) as a cofactor for deacetylation of *ε*-N-acetyl-lysine substrates, and are considered NAD^+^-dependent [9]. In this review, zinc-dependent or “classical” HDACs will be considered due to the target activity of the hydroxamic inhibitors discussed in the review.

In addition to enzymatic activity, HDACs have functional activity, which consists of gene expression through direct interaction with transcription factors. For example, HDACs can engage a number of different corepressors that inactivate target transcription factors p53, STAT3, E2F1, NF-κB, etc. [10,11].

Increased expression of HDACs of various classes in pathologies in various organs determines the extensive biological activity of compounds for which HDACs act as a therapeutic target. In particular, HDAC1 increases the expression of anti-inflammatory cytokines in inflammatory diseases of the oral cavity [12] and glioblastoma [13], HDAC1 and HDAC3 are hyperexpressed in cardiomyocytes in cardiomyopathy [14,15], and HDAC2 regulates the activation of hepatic stellate cells in biliary atresia [16].

HDAC inhibitors are able to improve tissue repair in various organs (brain, heart, liver, lungs, skin, muscles), which determines their great therapeutic potential in the study and treatment of diseases or conditions alleviated by modulating HDAC activity. For example, the HDAC6 isoform is expressed predominantly in the heart, brain, skeletal muscles, and skin, the HDAC10 isoform is expressed in the liver, and the HDAC11 isoform is expressed in smooth muscles, heart, kidneys, and brain tissue [17,18].

**Figure 2:**
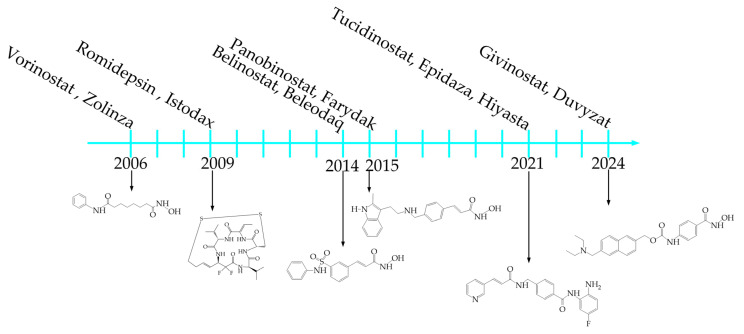
Timeline for commercial HDAC inhibitors.

To date, four compounds belonging to the HDAC inhibitors have been approved as anti-cancer drugs by the US Food and Drug Administration (FDA). These include vorinostat (SAHA, Zolinza), belinostat (Beleodaq), panobinostat (Farydak), and romidepsin (Istodax). The first commercial inhibitor approved by the US FDA for the treatment of T-cell lymphoma was vorinostat, which belongs to the class of hydroxamic acids, in 2006 [19]. In 2009, romidepsin, which belongs to the peptide class, was approved for the treatment of T-cell lymphoma, and belinostat was approved in 2014. In 2015, panobinostat was approved for the treatment of multiple myeloma. In 2021, the Japanese FDA approved tucidinostat (Epidaza, Hiyasta) for the treatment of relapsed T-cell leukemia. In 2024, givinostat (Duvyzat) was approved for the treatment of Duchenne muscular dystrophy [20] (Figure 2).

HDAC inhibitors can be used as monotherapy [21] or in combination with other treatments such as chemotherapy with gemcitabine, oxaliplatin, or dexamethasone [22,23], radiation therapy [24], dual targeted therapy [25], nanoscale polymer targeting therapy [26], and immunotherapy [27] to improve treatment efficacy and overcome drug resistance.

However, monotherapy using nanomolar concentrations of commercial HDAC inhibitors is effective only in some tumor types, mainly T-cell lymphoma and multiple myeloma [28,29]. In addition, commercial HDAC inhibitors such as vorinostat have poor selectivity, which leads to the death of both cancer and normal cells and numerous side effects such as anemia, asthenia, nausea, fatigue, thirst, pneumonia, and deep vein thrombosis [30,31]. Side effects of romidepsin include T wave flattening, hematological toxicity, thrombocytopenia, neutropenia, and anemia [32].

It should also be noted that HDAC inhibitors that are non-selective to a specific HDAC isoform or isoforms are less promising therapeutic agents in monotherapy and combination therapy compared to inhibitors specific to an HDAC isoform/isoforms, as they may exhibit undesirable systemic side effects [33]. Therefore, medicinal chemists are currently faced with the task of developing a structural design for HDAC inhibitors that are selective and less toxic to normal cells, which requires an integrative approach to understanding the structure and dynamics of protein–ligand interactions and assessing the effect of new HDAC inhibitors on the activity of various HDAC isoforms.

To date, reviews have been published devoted to either monotherapy of cancer, lung diseases (HDAC3), using HDAC inhibitors [34,35,36] and the effect of HDAC inhibitors on the immune system [37], or the antitumor activity of commercial chemotherapy drugs, for example, cisplatin and other platinum-based drugs [38,39]. The present review is devoted to the analysis of publications over the past two years in the field of HDAC inhibitors intended for the monotherapy of not only cancer but also neurodegenerative diseases (Alzheimer’s disease, Parkinson’s disease), diseases of the cardiovascular system, liver, lungs, and psoriasis. The review examines the dependence of the activity of HDAC inhibitors on their structure and provides recommendations for studies that are necessary for their introduction into clinical practice (in vivo studies).

## 2. Analysis of Publication Activity

**Figure 3:**
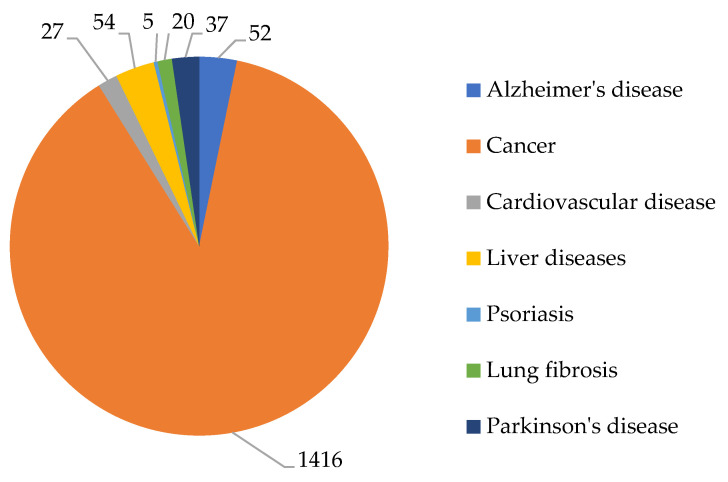
Number of articles for 2020–2024.

According to the Scopus abstract scientific database, the development of new HDAC inhibitors is a relevant area of research. The greatest interest over the past five years (2020–2024) has been observed in the field of developing new HDAC inhibitors as drugs for cancer therapy (1416 articles excluding review articles). In second place, in terms of publication activity, are works devoted to the use of HDAC inhibitors for the treatment of neurodegenerative diseases (Alzheimer’s disease and Parkinson’s disease), in third place are works on the treatment of liver diseases, and in fourth place are cardiovascular diseases and lung diseases (in particular, pulmonary fibrosis). In turn, the smallest number of works are devoted to the treatment of autoimmune diseases, such as psoriasis (Figure 3).

In our opinion, the higher publication activity in the field of using HDAC inhibitors for the treatment of oncological diseases compared to other pathologies is due to the fact that histone deacetylation is one of the main mechanisms of tumor development, contributing to resistance to antitumor agents. HDAC enzymes are associated with several aspects of cancer, such as cell cycle regulation, resistance to apoptosis, and interaction with the tumor microenvironment [40]. In addition, drugs developed over the past twenty years for the treatment of oncological diseases, in particular proteasome inhibitors, immunomodulatory drugs, monoclonal antibodies, targeted drugs, and peptide–drug conjugates, have shown resistance and a high risk of relapse in patients [40].

In the case of Alzheimer’s disease, which has a complex etiology, the mechanism of tau protein phosphorylation and accumulation of neurotoxic β-amyloid Aβ aggregates [41] with the participation of HDAC is not the main one. It is additionally based on such factors as acetylcholine deficiency, neuroinflammation, oxidative stress, biometal dyshomeostasis, glutamate imbalance, insulin resistance, intestinal microbiome abnormalities, cholesterol homeostasis disorder, mitochondrial dysfunction, and autophagy abnormalities [42]. For cardiovascular diseases and syndromes, in particular hypertension, β-adrenergic receptor antagonists and calcium channel blockers have proven themselves well [43].

**Figure 4:**
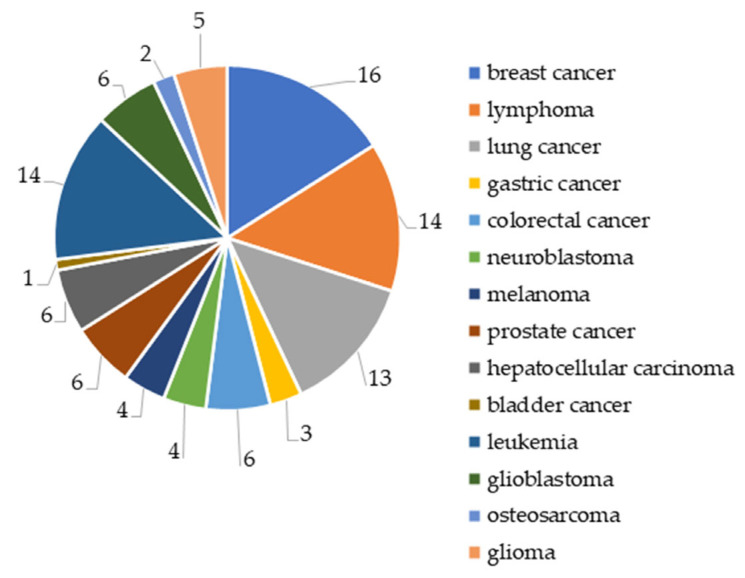
Share of total number of articles on cancer therapy for 2020–2024 (%).

Among tumor diseases, the greatest interest for researchers is breast cancer (16% of the total number of publications), leukemia (14%), lymphoma (14%), and lung cancer (13%), and the least interest is in bladder cancer, osteosarcoma, and gastric cancer (1–3%) (Figure 4).

In our opinion, the largest number of articles on breast cancer therapy is associated with scientists’ attempts to obtain a drug selective to HDAC isoforms that would pass clinical trials, unlike vorinostat. In turn, a large number of works on lymphoma therapy are associated with the need to minimize the side effects of the commercial drugs vorinostat and belinostat used to treat progressive T-cell lymphoma after systemic therapy.

**Figure 5:**
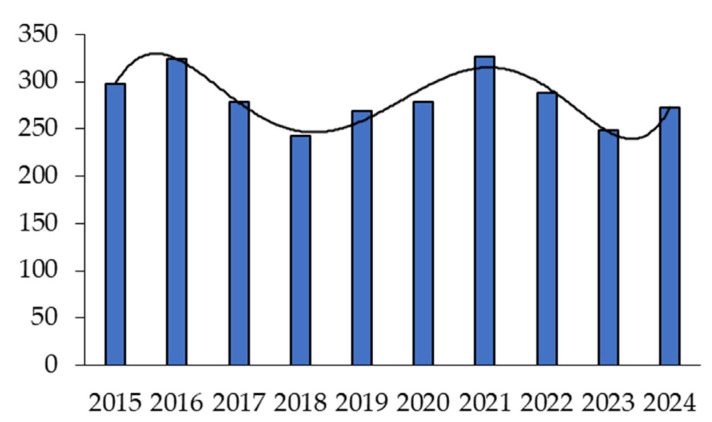
Number of publications devoted to HDAC inhibitors for cancer therapy with power law approximation.

In turn, the distribution of publications devoted to studies of HDAC inhibitors for the treatment of tumor diseases over the past ten years has shown two characteristic waves (Figure 5).

The greatest interest in HDAC inhibitors was observed in 2016 and 2021 and the least in 2018 and 2023. In 2024, there was a renewed interest in HDAC inhibitors. The increase in publications in 2021 may be due to the approval of tucidinostat in Japan. This drug has not been approved in the United States and requires additional randomized clinical trials. The decline in interest in developing new drugs for cancer therapy in 2019 is likely due to the onset of the COVID-19 pandemic.

## 3. General Structure of HDAC Inhibitors

**Figure 6:**
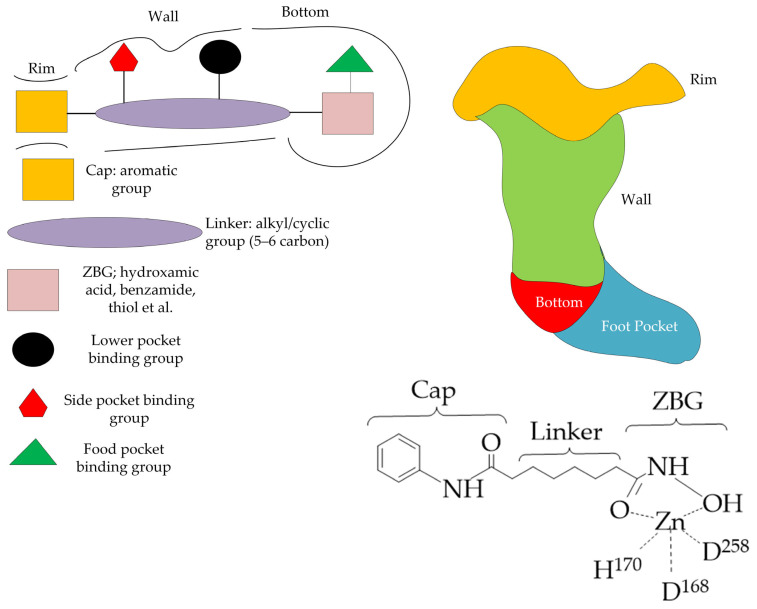
Schematic representation of the structure of HDAC inhibitors and image of HDAC class IIa selective bonding site and pharmacophore model using the example of the commercial HDAC inhibitor vorinostat (D^168^ and D^258^ are aspartic acid residues, and H^170^ is a histidine residue).

Most HDAC inhibitors, in particular hydroxamic acids and benzamides, bind to the main pocket of the enzyme’s catalytic center and chelate the zinc ion located at its bottom. The classical pharmacophore model includes three elements: a zinc binding group (ZBG) coordinating the catalytic zinc ion, a linker connecting the ZBG, and a cap group interacting with amino acid residues at the entrance to the enzyme’s active center (Figure 6). The extended structure of an HDAC inhibitor can include up to six elements, in particular additional binding groups of the side pocket, bottom pocket, and foot pocket [44].

The ZBG group of HDAC inhibitors increases the binding affinity to the enzyme and affects the selectivity of HDAC inhibition. Some of the ZBG groups demonstrate selectivity for binding to one or another HDAC isoform. For example, HDAC inhibitors in which aminoanilides, hydrazides, benzamides, and imidazolethiones act as ZBG groups are highly selective for class I HDAC isoforms (HDAC1, HDAC2, HDAC3, HDAC8) [45,46]. However, most ZBGs (hydroxamic and carboxylic acids, thiols, epoxy ketones, alkyl and aryl ketones) do not have high specificity [47]. Selectivity for HDAC inhibitors with these ZBGs is achieved by varying the linker and cap group.

**Figure 7:**
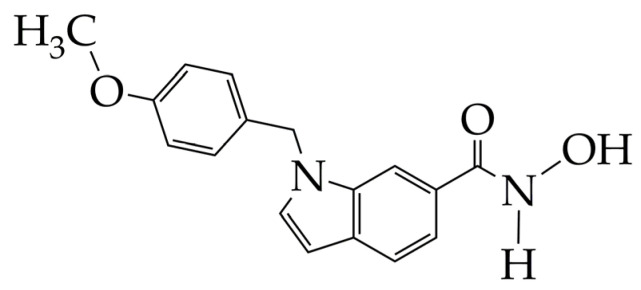
Chemical structure of PCI-34051 (PCI-34051 is at the preliminary clinical trial stage).

Commercial non-selective inhibitors (SAHA, belinostat, panobino-stat) typically contain alkyl and vinyl linkers. In turn, compounds containing five- and six-membered ring linkers demonstrate selectivity for HDAC6 or HDAC8. Hydroxamic acid PCI-34051 (Figure 7) is a selective inhibitor of HDAC8. PCI-34051 has a non-linear but ring linker in its structure, which specifically interacts with the catalytic pocket of HDAC8, namely with tyrosine Y306 and loops L1 and L6 of HDAC8 [48]. Hydroxamic acid PCI-34051 showed the induction of a lower energy state of the protein–ligand system compared to SAHA, which indicates its better inhibitory effect. This is due to the fact that, unlike SAHA with a flexible hydrocarbon linker, PCI-34051 with a rigid linker forms a stronger interaction and more intensively stabilizes the energy state.

For the cap group, it is quite difficult to predict the structure–selectivity relationship. Often, natural and synthetic compounds with bulky cap groups (e.g., tubacin) exhibit selectivity towards class IIb HDACs (HDAC6, HDAC10) [47]. Benzamides with a pyridine fragment as a cap group are selective towards the HDAC3 isoform [49].

A promising strategy for improving the selectivity of HDAC inhibitors is the modification of hydroxamic acids with cap groups of natural compounds, such as adamantane and terpene compounds [50,51,52]. For example, in [50,51], selective HDAC6 inhibitors were synthesized, containing fragments of adamantane or natural terpene compounds camphane, fenchane, and pinane as cap groups. The authors showed that hydroxamic acid with an adamantane moiety linked by an amide bond to a carbohydrate linker effectively inhibits HDAC6 by forming hydrogen bonds with HIS610, HIS651, and TYR782 of the catalytic center of HDAC6 and hydrophobic interactions with PRO608, PHE620, PHE679, PHE680, LEU749, and EHK782 and is a potential agent against Alzheimer’s disease.

In turn, hydroxamic acids containing a bicyclic pinane framework in the cap group inhibit HDAC1 of tumor cells, due to their high affinity for HDAC1, due to the formation of hydrogen bonds with the enzyme allosteric center [52]. The resulting hydroxamic acids with a bicyclic pinane framework are effective for eradicating tumor cells and overcoming drug resistance in the treatment of malignant neoplasms.

## 4. HDAC Inhibitors in Cancer Therapy

Cancer is the second leading cause of death, accounting for approximately 10 million deaths worldwide [53]. The high mortality rate from cancer is associated with the complexity of its therapy, due to its heterogeneity, numerous genetic and epigenetic changes, rapid development of resistance to treatment, and complex network of cellular signaling pathways that contribute to its malignancy [54,55]. Therefore, the development of new approaches to cancer therapy and the design of new drugs is a relevant area of research.

### 4.1. Breast Cancer

Breast cancer is the most common cancer in women in 157 of 185 countries, accounting for 2.3 million deaths per year [56,57]. This determines the highest interest in breast cancer among researchers compared to other types of cancer.

Triple-negative breast cancer (TNBC), which is a subtype of breast cancer, is the most aggressive of the subtypes (in particular, invasive ductal carcinoma) and is associated with a poor prognosis and low five-year survival of patients [58]. Available chemotherapeutic drugs for the treatment of TNBC, such as doxorubicin, epirubicin, paclitaxel, and others, have high cytotoxicity towards normal cells, and the current therapeutic options (chemotherapy and endocrine therapy) for its treatment are ineffective [59,60,61].

Data on the epigenetic modifications in the metabolic regulation in TNBC [62] and high activity of the HDAC6 isoform in non-meningeal TNBC cells [63] make the development of selective HDAC6 inhibitors for TNBC therapy a promising direction.

In [64], a series of hydroxamic acid derivatives containing the 1.3.8-triazaspiro[4.5]decan-4-one cycle as a cap group were synthesized. The resulting compounds acted as potent and selective inhibitors of HDAC6, as evidenced by the order of magnitude lower IC_50_ values of HDAC6 compared to HDAC1 (Table 1). In addition, micromolar (in the range of 25–27 μM) concentrations of compounds containing two fluorine atoms in the aromatic linker showed a highly effective antiproliferative effect on MDA-MB-231 TNBC cancer cells in the MTT test.

The selectivity of the obtained compounds in relation to HDAC6 is explained by the authors by the ability of HDAC6, unlike other HDAC isoforms, in particular HDAC1, to accommodate bulky and hydrophobic cap groups in the L1 pocket (D497-P501).

However, the authors of [64] did not investigate in vivo pharmacokinetic profiling for the synthesized hydroxamic acids, which are necessary for clinical trials involving humans. The importance of pharmacokinetic studies is due to the fact that if an HDAC inhibitor has a poor pharmacokinetic profile, then it will also have low bioavailability and undesirable toxicity.

**Figure 8:**
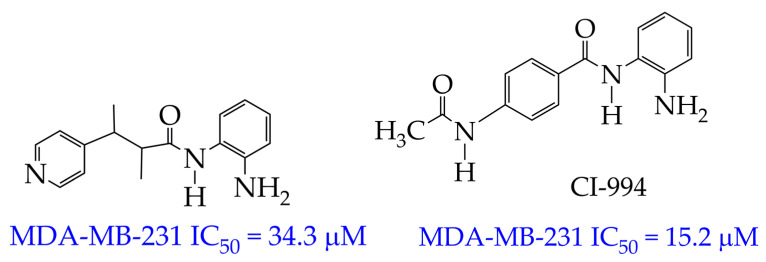
Chemical structure of N-(2-amino phenyl)-2-methyl-3-(pyridine-4-yl) butanamide and CI-994 (CI-994 is at the preliminary clinical trial stage).

In the work [65], not only were selectivity and cytotoxicity studies towards the MDA-MB-231 cell line conducted, but the pharmacokinetic profile in vivo was also studied for the HDAC inhibitor belonging to the benzamide class (Figure 8). The pyridine group served as the cap group in these compounds, and the chiral methylene fragment served as the linker. Pharmacokinetic studies were carried out by intraperitoneal administration at a dose of 25 mg/kg to male SD rats. For this, the benzamide was dissolved in a saline solution containing 5% DMSO and 30% polyethylene glycol. As a result, a higher value of the maximum concentration in blood plasma at T_max_ 2 h (C_max_ 16.22 μg/mL) was obtained compared to the previously synthesized benzamide CI994 (C_max_ 12.39 μg/mL). The obtained results indicated the good bioavailability and pharmacokinetic profile of benzamide at a low dose.

In addition to the pharmacokinetic data, the antitumor activity in vivo in female Balb/c mice with breast cancer was assessed in [65]. The authors found a lower growth in tumor volume (from 115 to 223 mm^3^) over 21 days of observation under the influence of 25 mg/kg of the synthesized benzamide compared to CI-994 (growth from 121 to 839 mm^3^).

**Figure 9:**
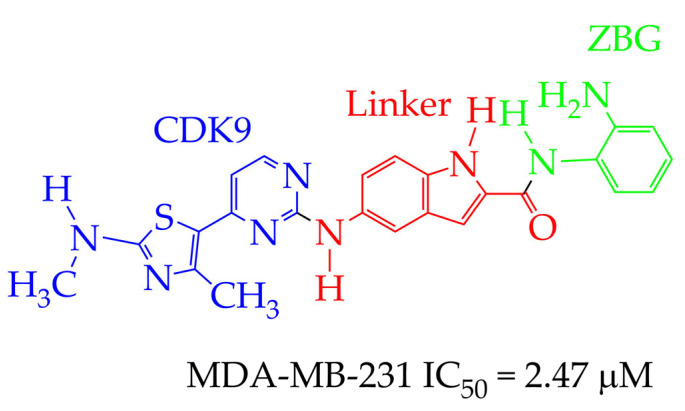
Chemical structure of N-(2-aminophenyl)-5-((4-(4-methyl-2-(methylamino)thiazol-5-yl)pyrimidin-2-yl)amino)-1H-indole-2-carboxamide.

In contrast to the HDAC inhibitor monotherapy discussed in [65,66], in our opinion, a more promising direction is combinatorial transcriptional therapy aimed at several targets. In [66], the cytotoxic effect of a dual-functional inhibitor acting on both class I HDAC (HDAC1 and HDAC3) and cyclin-dependent kinase 9 (CDK9), which is an important activator of transcriptional regulation, was investigated in relation to MDA-MB-231 cancer cells. CDK9 maintains transcription of RNA of anti-apoptotic proteins Bcl-2, Mcl-1, and XIAP at high levels [67]. The authors of [66] established a strong anti-proliferative activity of N-(2-aminophenyl)-5-((4-(4-methyl-2-(methylamino)thiazol-5-yl)pyrimidin-2-yl)amino)-1H-indole-2-carboxamide (Figure 9) against MDA-MB-231 cell lines. The IC_50_ value of the synthesized inhibitor was 2.47 μM. The o-aminoanilide group acts as the ZBG group in the obtained compound, and the 2-aminopyrimidine and aminothiazole fragments participate in the inhibition of CDK9.

In turn, in vivo analysis showed significant inhibition of tumor growth in the mouse xenograft MDA-MB-231 under the action of 30 mg/kg of the synthesized compound. This was evidenced by a decrease in tumor volume after 10 days of observation from 800 mm^3^ for the control group (saline solution administered intraperitoneally daily) to 200 mm^3^. The tumor growth inhibition rate at a concentration of 30 mg/kg was 77%.

**Figure 10:**
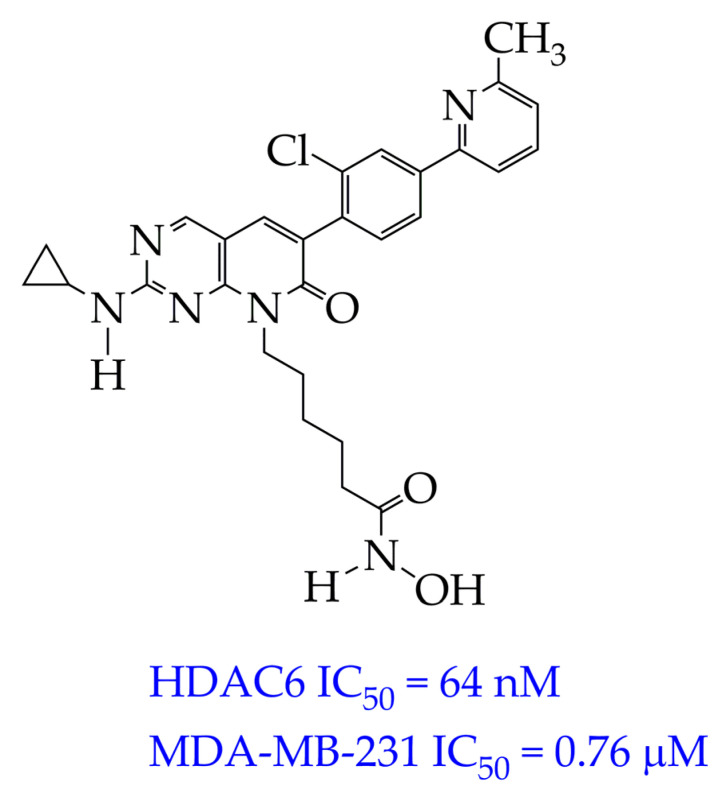
Chemical structure of ZMF-25 (ZMF-25 is at the preliminary preclinical studies stage).

In addition to CDK9, an important therapeutic target in TNBC is the serine/threonine-protein kinase PAK1, which is involved in the management of oncogenesis, angiogenesis, and metastasis and plays a role in resistance to therapy. The authors of [68] synthesized a dual-targeting inhibitor of PAK1 and HDAC6/HDAC10, which is able to overcome the heterogeneity of TNBC. The inhibitor is pyrido[2,3-d] pyrimidin-7(8H)-one-coupled hydroxamic acid (ZMF-25, Figure 10). ZMF-25 showed high activity against PAK1 and HDAC10 in the nanomolar HDAC range (33 nM PAK1, 64 nM HDAC6, and 41 nM HDAC10) and a potent antiproliferative effect on 0. cells (IC_50_ 0.76 μM).

The authors explain the inhibition of PAK1 by the presence of an aminopyrimidine group in the ZMF-25 structure, which forms hydrogen bonds with the Leu^347^ residue located in the kinase hinge. In turn, the hydroxamic acid fragment (ZBG group) and 6-phenylpyrido[2,3-d]pyrimidin-7(8H)-one core (cap group) are involved in the inhibition of HDAC6 and HDAC10.

It is noteworthy that ZMF-25 promoted the generation and accumulation of reactive oxygen species (ROS) by inhibiting PAK/HDAC6/10, thereby impairing mitochondrial function, which resulted in a significant decrease in ATP production and a marked decrease in mitochondrial membrane potential. Induction of autophagy, decreased expression of glycolytic genes, and impaired mitochondrial metabolism with increased metabolic stress were also observed.

However, despite the potent antiproliferative effect of ZMF-25 and the promising prospects for its use in the treatment of TNBC, further studies are required on its toxicity to normal cells, in particular MCF-12A breast epithelial cells.

As noted in Section 2, the second place after breast cancer in terms of publication activity is occupied by a tumor formed by lymphocytes—lymphoma.

### 4.2. Lymphoma

Despite significant progress in the treatment of lymphomas, due to the development of new chemotherapy and biological therapy methods, a significant number of lymphomas are prone to relapse and refractory problems [69].

Currently approved for the treatment of T-cell lymphoma in patients who do not respond to other treatments, vorinostat has many side effects [70]. In our opinion, to minimize side effects, it is necessary to develop a drug that would be multitargeted.

The use of multi-targeted drugs is a successful strategy in the treatment of lymphomas. In the treatment of lymphomas, it is of great importance to target the drug not only to HDAC but also to phosphatidylinositol-3-kinase (PI3K), which is involved in the PI3K/Akt/mTOR signaling pathway. This signaling pathway is activated in Hodgkin’s and non-Hodgkin’s lymphomas and increases metastatic ability and resistance to traditional therapy [71].

**Figure 11:**
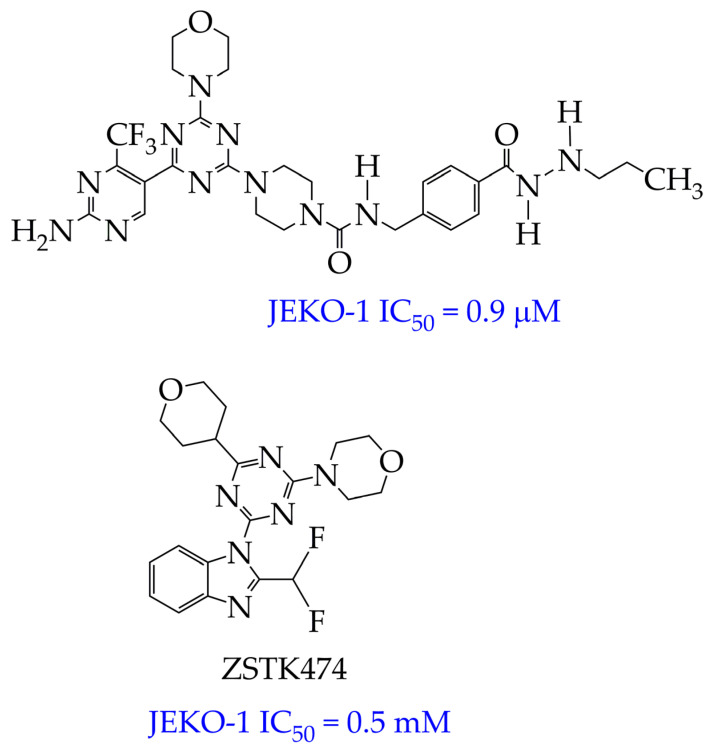
Chemical structure of 4-(4-(2-amino-4-(trifluoromethyl)pyrimidin-5-yl)-6-morpholino-1,3,5-triazine-2-yl)-N-(4-(2-propylhydrazine-1-carbonyl)benzyl)piperazine-1-carboxamide and ZSTK474 (ZSTK474 is at the preliminary clinical studies stage).

In the work [72], a dual PI3K/HDAC1-3 inhibitor belonging to the hydrazide class was synthesized (Figure 11). Dual inhibition was due to the presence of a morpholine-triazine scaffold targeting PI3K and a hydrazide fragment as a ZBG group. It showed good antiproliferative activity against mantle cell non-Hodgkin’s lymphoma JEKO-1 cells (IC_50_ 0.9 μM), which was superior to the activity of the previously synthesized [73] single-target PI3K inhibitor ZSTK474 (IC50 1.8 μM). The dual inhibitor at a concentration of 0.5 μM induced approximately 47% apoptosis in JEKO-1 cells, outperforming ZSTK474 (only 25%).

In addition, the synthesized PI3K/HDAC1-3 inhibitor did not exhibit significant cytotoxicity (IC_50_ greater than 100 μM) against the normal human hepatocyte cell line HL7702, which indicates the selectivity of its antitumor effect and the relevance of further in vivo studies with its participation.

**Figure 12:**
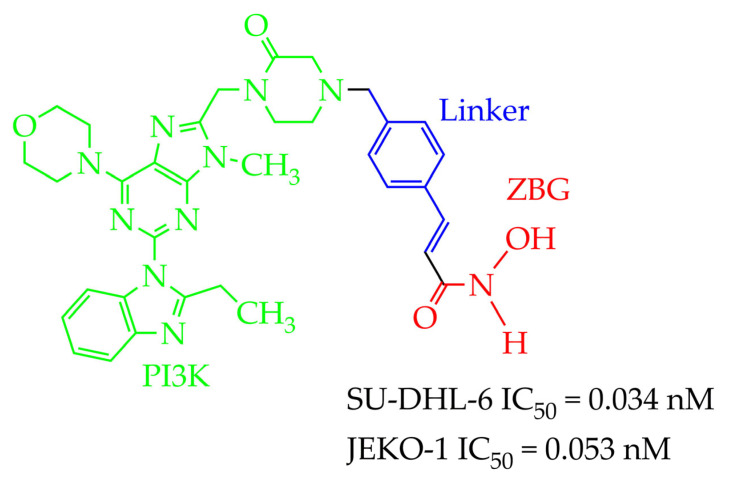
Chemical structure of novel PI3K/HDAC6 dual inhibitors.

In addition to hydrazides, hydroxamic acids are also considered dual-targeting inhibitors for lymphoma therapy. In [74], hydroxamic acid (Figure 12) demonstrated potent inhibitory activity against HDAC6 and PI3K and antiproliferative activity against multiple non-Hodgkin’s lymphoma (NHL) cells, diffuse large B-cell lymphoma SU-DHL-6 cells, and mantle cell lymphoma JEKO-1 cells (IC_50_ value less than 0.1 µM). The methylene moiety between the aromatic linker and piperazine contributed to the flexibility of the compound and improved inhibitory activity against HDAC6. In addition, hydroxamic acid (25 mg/kg) prevented tumor growth in the SU-DHL-6 and JEKO-1 xenograft model.

The authors also showed that replacing the hydroxamic moiety with an o-phenylenediamine moiety resulted in a loss of activity against HDAC6 (IC_50_ increases from 6.2 nM to 1000 nM). However, the authors of the work [74] did not provide an explanation for this fact. In our opinion, this is due to the absence of a hydrogen bond with Gly^582^, which is characteristic of hydroxamates, according to molecular docking data.

In our opinion, dual-targeting inhibitors based on hydroxamic acids are more promising compounds for the treatment of non-Hodgkin’s lymphomas compared to hydrazides, since they show lower IC_50_ values (<0.1 μM, for hydrazides > 0.1 μM) in the study of antiproliferative action against JEKO-1 cells. However, in the future, it is necessary to consider the possibility of combining the considered dual-targeting inhibitors with various chemotherapeutic drugs, which will allow the creation of new treatment regimens with a high percentage of five-year survival.

### 4.3. HDAC Inhibitors for Other Cancers

HDAC inhibitors belonging to the hydroxamic acid class, in addition to breast cancer and lymphomas, are promising compounds for the treatment of melanoma [75], acute myeloid leukemia, leukemia, colorectal cancer, and gastric cancer. Hydrazides can also be used for the treatment of melanoma. However, hydrazides have shown low exposure when taken orally and low oral bioavailability [76]. Table 2 shows the structural formulas of some HDAC inhibitors used for cancer therapy in studies over the past two years and summarizes the results obtained in these studies.

Based on the chemical structure of the HDAC inhibitors presented in Table 2, it can be concluded that the greatest interest for further in vivo studies and clinical trials is HDAC inhibitors of the hydroxamic acid class containing 2-(4-(diphenylamino)benzylidene)hydrazinyl [75], 2-(3-(trifluoromethyl)phenyl)ethynyl [77], and (4-methylphenyl)carbamoylmethoxy fragments [78] as a cap group.

**Figure 13:**
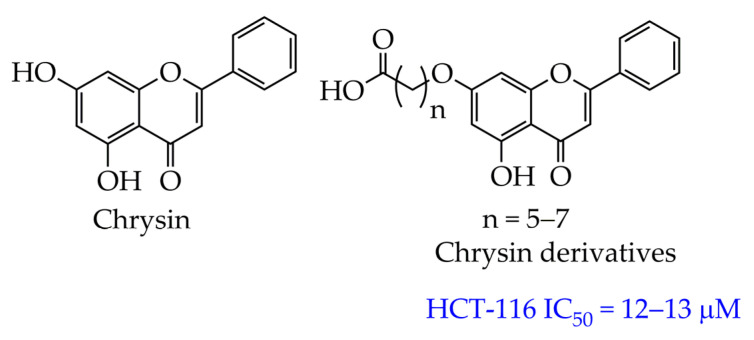
Structures of chrysin and synthetic chrysin derivatives.

In addition to hydroxamic acids, synthetic analogs of secondary plant metabolites belonging to the class of flavonoids are promising compounds for inhibiting HDAC (Figure 13). In particular, a synthetic derivative of chrysin (5,7-dihydroxyflavone), contained in the Indian tubular flower *Oroxylum indicum*, has a selective effect on the HDAC8 isoform and an antiproliferative effect against the HCT-116 colon cancer cell line (IC_50_ 12–13 μM) [83]. Its inhibitory effect is associated with the formation of a zinc carboxylate complex and the coordination of hydroxyl -OH, formyl C=O groups of the flavonoid with the zinc ion Zn^2+^. In addition, this compound has lipophilic properties (ClogP ≤ 5), which allows it to be administered not only intravenously but also orally.

Most studies on HDAC inhibitors for cancer therapy consider the inhibitory effect on a specific HDAC isoform and the in vitro antiproliferative effect against cancer cell lines (15 studies), but the cytotoxicity to normal cells, apoptosis, inhibition of migration, and in vivo studies (6 studies) are considered in fewer studies. In turn, in vitro studies have a number of limitations, since they are conducted in a controlled environment without the complex interactions that occur in a living organism. They do not take into account systemic factors such as the influence of the immune system, metabolism, and interaction with other organs. In addition, the culture media, temperature, and environment used may not fully reflect physiological conditions, which leads to a discrepancy with the potential behavior of the substance in a natural physiological environment. Therefore, to conduct clinical trials of the considered HDAC inhibitors, it is necessary to study their own antitumor effect in vivo using model laboratory animals (rats, mice, pigs, rabbits, etc.) with grafted tumors, as well as to study the pharmacokinetics in vivo, which allows us to understand how the compound is absorbed, distributed, metabolized, and excreted from the body. It is also necessary to determine the toxicity class of the compounds, study the chemosensitizing activity of the compounds with known cytostatics in model animals, determine the degree of metastasis in animal organs, and measure the tumor size.

In addition to antitumor action, another pharmacological action of HDAC inhibitors is neuroprotective activity, making them promising compounds for the treatment of neurodegenerative diseases.

## 5. HDAC Inhibitors for the Treatment of Nervous System Diseases

### 5.1. Alzheimer’s Disease

Alzheimer’s disease (AD) is an irreversible and progressive neurodegenerative disease affecting the nervous system with a multifaceted pathophysiology [84]. AD is considered to be the most common neurodegenerative disease, accounting for 60–70% of dementia cases worldwide [85]. Class I isoforms of HDAC1, HDAC2, and HDAC3 are thought to be mainly involved in memory dysfunction in AD, while downregulation of HDAC6, which regulates microtubule function and stability through tubulin acetylation, promotes clearance of tau protein and beta-amyloid Aβ [86]. These HDAC isoforms are overexpressed in the brain of AD patients, primarily in the entorhinal cortex and hippocampus [87].

**Figure 14:**
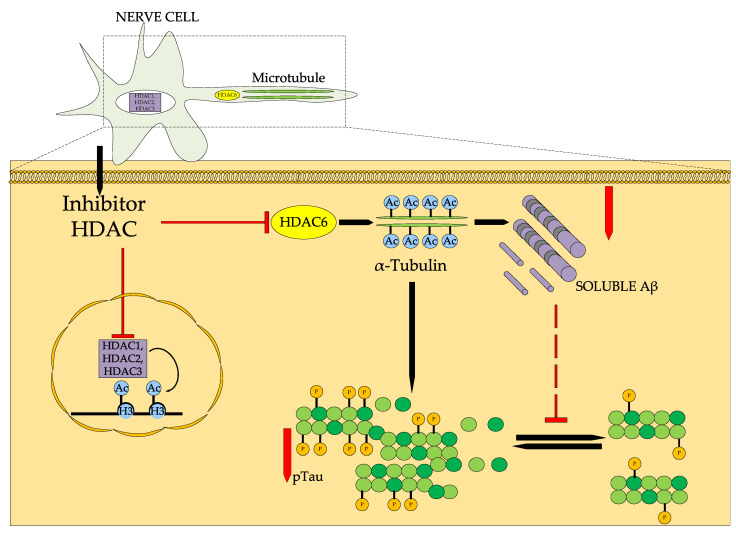
Mechanism of action of HDAC inhibitor Ac—acetyl, P—phosphoric acid.

Figure 14 shows the general model of HDAC inhibitors in Alzheimer’s disease. HDAC inhibitor can inhibit both HDAC1, HDAC2, and HDAC3 activity in the neuronal nucleus, thereby increasing the levels of acetylated histone H3 protein and improving transcription, and HDAC6 activity in the neuronal axon, promoting the degradation of misfolded non-histone proteins such as tau protein and Aβ, suppressing tau protein hyperphosphorylation [88].

A promising direction for AD therapy is therapy using epigenetic agents—HDAC inhibitors (Table 3). For example, treatment with bufexamac (20 μg/kg), which belongs to the class of hydroxamic acids, improves cognitive and behavioral impairments in rats administered Aβ for 8 days. Bufexamac has a therapeutic advantage over previously studied drugs such as tubastatin A [89].

In addition to bufexamac, hydroxamic acid containing a phenothiazine moiety as a cap group and a pyridine ring as a linker (Table 3) is an effective and selective inhibitor of HDAC6. It increases the levels of acetylated α-tubulin in SH-SY5Y cells, which have a similar morphology to mature neurons, and reduces the hyperphosphorylation of tau protein [90].

However, in some cases, HDAC inhibitor monotherapy is ineffective. This is due to the multifactorial nature of AD. In addition to HDAC, glycogen synthase kinase-3β (GSK-3β) is involved in the phosphorylation of tau protein and plays a key role in modulating susceptibility to ferroptosis [91].

Lithium chloride is known to be an effective inhibitor of GSK-3β [92]. The synergistic neuroprotective effect of WY118 and lithium chloride was investigated in this work [93]. The authors found that the viability of HT-22 mouse hippocampal neuronal cells in a glutamate-induced model increased only with the combined effect of WY118 hydroxamic acid (0.8–2.5 μM) and lithium chloride (3.2–10 μM) from 5% to 84% after 24 h but not with WY118 monotherapy. In addition to neuroprotective activity, an in vivo study of metabolic stability in mice with cognitive deficit showed that the combination of WY118 and lithium chloride increased the half-life (158 min) compared to WY118 (110 min). This indicates an improvement in the metabolic stability of WY118 in the presence of lithium chloride. The authors also found an improvement in spatial learning and memory in the Morris Water Maze test.

Another aspect of AD pathogenesis is the disruption of the cholinergic system, manifested in a decrease in the biosynthesis of acetylcholine and excessive depletion of neurotransmitters. Butyrylcholinesterase (BChE), which hydrolyzes acetylcholine, plays a significant role in the regulation of cholinergic transmission and its activity is increased in patients with AD [94]. Butyrylcholinesterase inhibition may restore cholinergic function and improve cognitive function in patients with AD.

In [95], a dual target inhibitor of BChE/HDAC6 was developed that exhibited significant neuroprotective effects in vitro, as evidenced by the increased survival of HT-22 cells with peroxide-induced injury, and cognitive improvements in vivo in mice with an AD model (Table 3). In the structure of the obtained compound, the hydroxamic fragment and ethylene glycol linker were responsible for HDAC6 inhibition, and the amide bond was responsible for BChE inhibition. Thus, a multimodal therapeutic strategy is an effective way to inhibit the progression of AD.

However, clinical studies of HDAC inhibitors and dual inhibitors require studies of their effects on general behavioral characteristics in the Open Field test, effects on cognitive functions in the Novel Object Recognition and Morris Water Maze tests, as well as immunohistochemical analysis of β-amyloid deposits and assessment of antioxidant activity based on the ability to inhibit lipid peroxidation in rat brain homogenates in the modified TBA test (thiobarbituric acid test). Unfortunately, in the studies reviewed, only one study conducted the Morris Water Maze test, and the rest of the studies were not conducted.

**Table 3:** Chemical structures of HDAC inhibitors for Alzheimer’s disease treatment.

HDAC Inhibitors	Reference
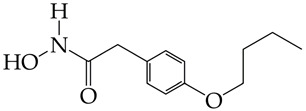 Bufexamac (DB13346), was also withdrawn by the EMA in April 2010	[89]
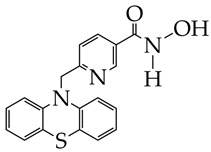 IC_50_ HDAC1 = 743 nM, IC_50_ HDAC2 = 1962 nM, IC_50_ HDAC6 = 2.54 nM	[90]
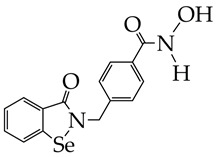 WY118 is at the preliminary preclinical studies stage.	[93]
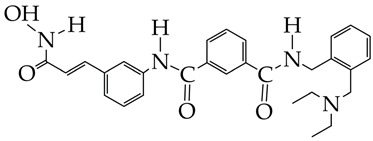 IC_50_ HDAC6 = 56.7 nM.	[95]

### 5.2. Parkinson’s Disease

Parkinson’s disease is a progressive neurodegenerative disorder with impaired motor function caused by the loss of dopaminergic neurons in the substantia nigra and characterized by the accumulation of α-synuclein. About 10 million people worldwide suffer from Parkinson’s disease, and by 2030, this number will increase by more than 50% [96].

L-DOPA, currently used to treat Parkinson’s disease, only temporarily alleviates symptoms and does not prevent disease progression [97]. Correction of the imbalance between histone acetylation and deacetylation using HDAC inhibitors is a promising strategy for the treatment of Parkinson’s disease. Restoring balance will result in neuroprotection by reversing mitochondrial dysfunction, suppressing ROS and inhibiting α-synuclein accumulation.

Benzamides and hydroxamic acids can be used as HDAC inhibitors in Parkinson’s disease. In [98], the HDAC inhibitor TMP269 (Table 4), which belongs to benzamides, prevented neutrino damage in SH-SY5Y cells induced by 6-hydroxydopamine. The use of micromolar concentrations of TMP269 increased the level of acetylated histones H3 and decreased HDAC5 activity in SH-SY5Y cells after 72 h of treatment. An increase in the expression of the neutrophic factor BMP2 was also observed after 24 h. An in vivo study demonstrated a decrease in nuclear HDAC5 in dopaminergic neurons in the substantia nigra of rats with 6-hydroxydopamine damage, which led to an improvement in motor function. In the work [99], hydroxamic acid LMK-235 inhibited HDAC5 and enhanced histone acetylation. LMK-235 protects SH-SY5Y cells from oxidative stress caused by cytosolic free dopamine.

However, the use of hydroxamic acid HDAC inhibitors, in particular CAY (Table 4), faces clinical limitations due to their poor water solubility and short biological half-life. To increase the half-life and significantly accumulate in the brain, a nanoparticle delivery system is used. Nanoparticles protect HDAC inhibitors from metabolism, reduce side effects, increase their ability to penetrate the blood–brain barrier, and control their release at the target site. For example, in [100], CAY loaded into poly(lactide-*co*-glycolide) PLGA nanoparticles demonstrated slower release kinetics and controlled release. The controlled release of CAY was associated with gradual hydrolysis of ester bonds in PLGA. Neuroprotective effects in vivo against methamphetamine-induced Parkinson’s disease were also found in C57BL/6J mice, as evidenced by improved locomotor performance and coordination.

Future studies should consider the effect of compounds on the microglia, determination of all biomarkers of Parkinson’s disease in the whole blood of mice, such as monoamines, urates, sphingolipids, microRNA, and lymphocytes, and determine the oxidative stress index, as well as the level of lipids isolated from the striatum and substantia nigra.

## 6. HDAC Inhibitors for the Treatment of Cardiovascular Diseases

Cardiac diseases remain a leading cause of global morbidity and mortality. Despite significant advances in the medical and surgical treatment of cardiac diseases, some patients suffer from myocardial dysfunction and structural damage even after therapy [101].

HDACs regulate gene expression in cardiac hypertrophy and are elevated in the development of heart failure after myocardial infarction [102].

**Figure 15:**
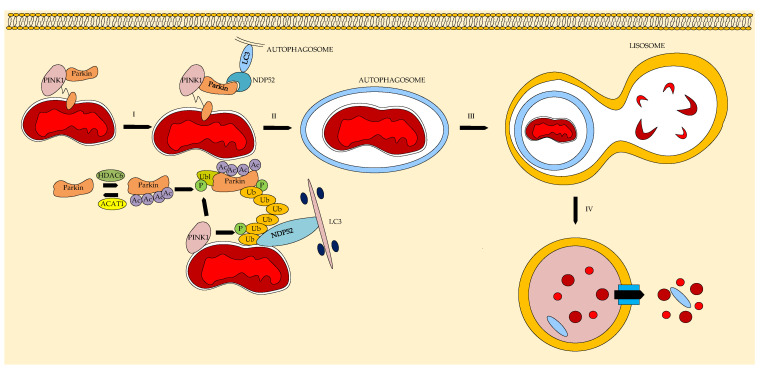
Autophagy process in H9c2 cardiomyocytes in heart failure (ACAT1—acetyltransferase 1, PINK1—putative kinase 1 or serine/threonine kinase, p—phosphoric acid, ub—ubiquitin, LC3—microtubule-associated proteins 1A/1B light chain 3, Parkin—protein with E3 ubiquitin-protein ligase activity, NDP52—nuclear dot protein 52, I—Parkin ubiquitinates targeted proteins and activates the autophagy-related protein NDP52 and anchors to the autophagosome with LC3 “grabber”, II—fusion of autophagosomes with damages mitochondria, III—autophagosome binds to lysosomes, IV—lysosomal degradation of damaged mitochondria.

Mitophagy is an evolutionary cellular process that protects cardiomyocytes during various myocardial injuries (e.g., infarction) [103]. HDAC inhibitors activate the acetylation of parkins, proteins involved in the PINK1/Parkin pathway that regulates mitophagy and the apoptosis of cardiomyocytes [104,105]. Figure 15 shows a diagram of mitophagy in a cardiomyocyte mediated by the PINK1/Parkin signaling pathway, which consists of removing damaged or dysfunctional mitochondria via the autophagy/lysosome pathway. PINK1 accumulates in the mitochondrial outer membrane during pathology and attracts parkin, which leads to ubiquitination to mark damaged mitochondria. Ubiquitinated parkin activates autophagy transition proteins such as nuclear dot protein 52 (NDP52) and anchors damaged mitochondria in autophagosomes by binding to microtubule-associated protein 1 light chain 3 (LC3), which is degraded in the lysosome.

In [106], it was shown that the inhibition of HDAC6 activity induced by isoproterenol in mice leads to an increase in the level of MAP1LC3B, which promotes autophagy and prevents myocardial hypertrophy in mice. Thus, the authors confirmed the involvement of the HDAC6 isoform in myocardial hypertrophy.

HDAC6 inhibitors can be used to treat dilated cardiomyopathy and chronic heart failure and prevent pathological cardiac hypertrophy.

Hydroxamic acid YAK577 attenuates cardiac fibrosis and improves cardiac function in heart failure by reducing H9c2 cardiomyocyte hypertrophy in vitro [107]. Varinostat (SAHA) inhibits HDAC activity, reduces glycolytic activity, and improves mitochondrial activity in dilated myocardial-isolated human myocardial mesenchymal stromal cells (hmMSC). This makes it a promising agent for the treatment of dilated cardiomyopathy [108]. However, YAK577 and SAHA do not have an antioxidant effect and do not affect the inhibition of oxidative stress observed in myocardial pathology. In addition, prolongation of the QT interval and an increase in the concentration of urea and creatinine have been reported with SAHA administration [109].

The use of a selenium-containing HDAC inhibitor (Se-SAHA, Table 5), which belongs to the benzamide class, made it possible to attenuate cardiac hypertrophy and fibrosis in ventricular myocytes of newborn rats, restore the expression levels of superoxide dismutase 2 and heme oxygenase 1 in vitro, and reduce the level of microtubule-associated protein LC3-II in mice with heart failure [110]. Furthermore, Se-SAHA prevented autophagosome accumulation, reversing isoproterenol-induced HDAC6 overexpression. The antioxidant effect of Se-SAHA is due to the presence of selenium in its structure.

Phytochemicals also have cardioprotective effects. As dietary compounds, they can be marketed without human testing, only in large animal models, under the Dietary Supplement Health and Education Act of 1994 (DSHEA), making research less expensive.

For example, emodin, which belongs to the class of anthraquinones and is found in rhubarb, cabbage, and beans, inhibits HDAC activity in cattle heart tissue and increases histone acetylation in cardiomyocytes [111]. Emodin also attenuates angiotensin II-induced cardiac fibrosis in male and female mice. Since emodin is of natural origin, it is likely to be safe for humans, unlike SAHA and Se-SAHA, and its consumption as a dietary supplement in low doses (a dose of 10 μM per 100 mg/L of rhubarb) will promote transcriptome normalization and improve cardiac muscle function. However, emodin has low bioavailability and requires the use of nanosized delivery systems (liposomes, nanoparticles).

In the field of drug development for the treatment of cardiac pathologies, the use of liposomal forms to improve their bioavailability and the use of a combination of compounds involved not only in the regulation of mitophagy but also in inhibiting the activity of free radical oxidation processes remain unexplored. For a comprehensive assessment of the cardioprotective effects of compounds, it is necessary to determine the biochemical markers of myocardial damage (cardiac troponin, aspartate aminotransferase, etc.) and conduct integral vector analysis of hemodynamic, biochemical, and morphological parameters and echocardiographic diagnostics in vivo.

## 7. HDAC Inhibitors for the Treatment of Liver Diseases

HDAC11, a member of the class IV HDAC subfamily and localized to the nucleus and cytoplasm, is overexpressed in metabolic dysfunction-associated steatotic liver disease (MASLD) [112]. MASLD is a dangerous pathology, associated with excess accumulation of fat in the liver, that can further develop into liver fibrosis, liver cirrhosis, and even hepatocellular carcinoma [113].

**Figure 16:**
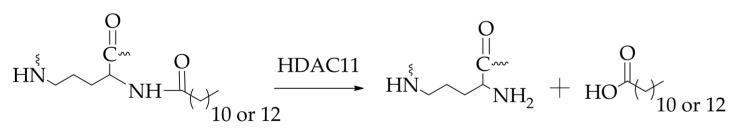
Reaction of hydrolysis of lysine residue by the enzyme HDAC11.

The authors of [112,114] explain this fact as follows: HDAC11 has a catalytic domain with a long cavity capable of accommodating long-chain fatty acyl groups and hydrolyzes lysine residues (Figure 16). HDAC11 is the only HDAC representative that is involved in the removal of long-acyl groups.

Inhibition of HDAC 11 promotes the inhibition of lipogenesis and promotes fatty acid oxidation in the liver. Cyclic peptides [115] and hydroxamic acids (PB94) [116] have been studied as HDAC11 inhibitors (Table 6).

However, their selectivity needs to be improved. In [112], hydrazide was used to improve the selectivity of the compounds (Table 6). To increase the hydrophilicity of the compound, an oxygen atom was introduced into the side carbon chain. Hydrazide demonstrated selectivity for HDAC11 (IC_50_ = 51.1 nM), since it had a strong affinity binding and did not affect the acetylation levels of histones H3 and H4 (substrates of class I HDAC1/2/3/8) (for HDAC1/2/3 IC_50_ > 10,000 nM, for HDAC8 IC_50_ = 4757 nM) or tubulin (substrate of HDAC6, IC_50_ > 10,000 nM). The authors explain this fact as follows: the chelation of zinc ions in HDAC11 by the hydrazide part and the incorporation of a long fatty tail into the hydrophobic cavity.

Hydrazide restored mitochondrial activity in the mouse hepatocyte line AML12, decreasing protein and mRNA expression of SREBP1c, which serves as a key transcription factor in the DNL metabolic pathway responsible for the synthesis of both saturated and monounsaturated fatty acids from acetyl-CoA. An in vivo study using the MASLD mouse model with oral administration of hydrazide showed a decrease in the liver weight and body fat mass. Biochemical analysis demonstrated a decrease in the level of alanine aminotransferase, aspartate aminotransferase, alkaline phosphatase, and cholesterol in the blood.

The resulting product is proposed for use in the treatment of MASLD. However, the exact mechanisms of MASLD regulation involving HDAC11 remain unclear and require further study. It is necessary to consider the involvement of not only the HDAC11 isoform in the pathogenesis of MASLD but also HDAC3, which regulates the activity of the transcription factor FoxO1 [117] and enhances the expression of fatty acid synthase genes. FoxO1 is involved in glucose homeostasis and gluconeogenesis in the liver and is of great importance for patients with obesity and diabetes mellitus, and fatty acid synthase catalyzes the synthesis of palmitate from acetyl-CoA and malonyl-CoA with the formation of saturated fatty acids.

## 8. HDAC Inhibitors for the Treatment of Pulmonary Fibrosis

Idiopathic pulmonary fibrosis (IPF) is a fatal lung disease with high mortality. Unfortunately, effective treatments for IPF are still lacking [118]. The FDA-approved drugs for the treatment of pulmonary fibrosis, nintedanib and pirfenidone, have shown little improvement in patient survival.

IPF is characterized by damage to the alveolar epithelium, initiating the production of cytokines and growth factors TGF-β and ET-1 (endothelin-1) [119]. HDACs in IPF catalyze the deacetylation of non-histone proteins, in particular MKP-1, thereby regulating their activity and downstream signaling pathways [120].

HDAC inhibitors, which belong to the hydroxamic acid class (Table 7), are potential candidates for the treatment of IPF. In the work [121], it was shown that the hydroxamic acid MPT0E028 induces acetylation and activation of MKP-1, inhibits phosphorylation of c-Jun N-terminal kinase (JNK), p38, and extracellular signal-regulated kinase (ERK) and suppresses the expression of CTGF (connective tissue growth factor) in human lung fibroblasts WI-38. In studies [122,123,124], hydroxamic acids containing imidazolepyrimidone, pyrrolo[2,1-c] [1,4]benzodiazepine-3,11-dione, and spirotetrahydroisoquinoline fragments, as cap groups inhibited HDAC6 activity. HDAC6 is overexpressed in pulmonary fibrosis, since it is the main deacetylase of α-tubulin. Hydroxamic acid with pyrrolo[2,1-c] [1,4]benzodiazepine-3,11-dione cap group at a concentration of 50 mg/kg did not have a negative effect on kidney function or toxic effects in a mouse model of IPF.

In the field of IPF therapy, the identification and validation of genes and the study of the level of markers of alveolitis and fibrosis (surfactant serum protein A) are needed to better understand the mechanisms of IPF pathology, as well as the use of combination drugs to regulate fibrotic and inflammatory reactions in the lungs.

## 9. HDAC Inhibitors for Psoriasis Therapy

Psoriasis is an inflammatory autoimmune skin disease characterized by a high rate of relapse after drug withdrawal [125]. Although significant advances have been made in the treatment of psoriasis through the development of immunosuppressants (thymodepressin) and neutralizing antibodies (basiliximab), relapses remain a serious problem, causing physical discomfort and emotional stress in patients [126,127]. A comprehensive understanding of the molecular mechanisms underlying this disease will allow for the development of more effective treatment strategies in the future.

**Figure 17:**
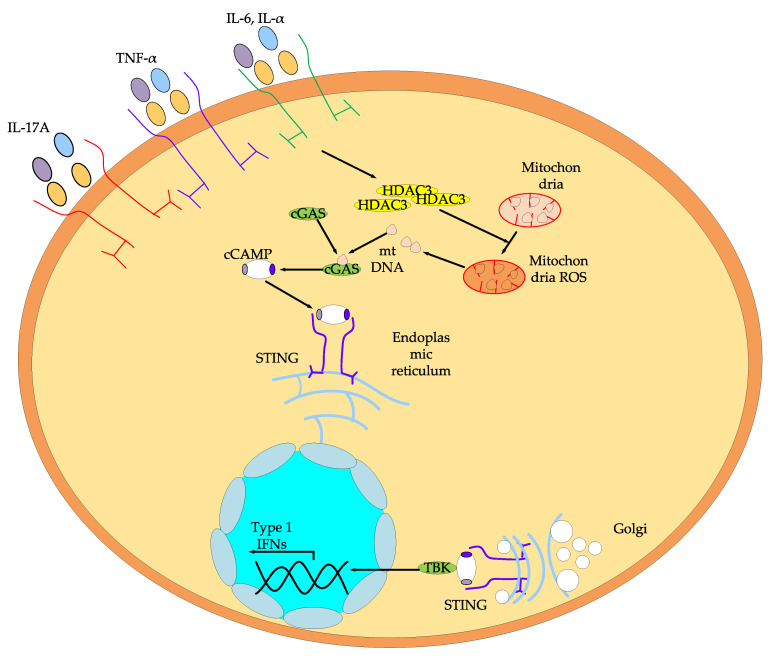
Scheme of inflammation regulation in keratinocytes.

HDACs are among the most important regulators of psoriasis. They modulate mitochondrial function and immune responses [128]. Increased expression of HDAC3 isoform mRNA in keratinocytes is observed in patients with psoriasis [129]. This is due to the fact that inflammatory cytokines induce HDAC3 overexpression in keratinocytes, which promotes ROS formation and structural damage to mitochondria. Mitochondrial damage releases mtDNA into the cytosol, triggering activation of the GMP–AMP synthase (cGAS)—stimulator of interferon genes (STING) pathway. As a result, the level of cGAS, STING, and phosphorylated TBK1 (p-TBK) proteins increases (Figure 17).

Zeng C. et al. [130] found that inhibition of the HDAC3 isoform using the specific inhibitor RGFP966 (Table 8), belonging to the class of benzamides, suppresses the activation of cGAS-STING signaling in HaCaT keratinocytes exposed to anti-inflammatory cytokines IL17A, TNF-α, IL-6, and IL-α.

Thus, inhibition of HDACs, in particular the HDAC3 isoform, alleviates mitochondrial dysfunction in psoriasis and impairs cytosolic DNA sensing and downstream signaling pathways.

In addition to HDAC, receptor tyrosine kinases—Janus kinases (JAK)—play an important role in the pathogenesis of psoriasis. Therefore, a more promising strategy for the treatment of psoriasis is dual inhibition of the activity of these enzymes. For example, Hu W. et al. synthesized a compound that inhibits both HDAC and JAK, in which a hydroxamic acid fragment is responsible for inhibition of HDAC, and an aminopyrimidine fragment is responsible for inhibition of JAK.

The obtained compound demonstrated excellent antipsoriatic effects in a mouse model of imiquimod-induced psoriasis. The compound attenuated the pathological symptoms in mice, as evidenced by a decrease in the thickness of the skin epidermis [131]. Thus, dual JAK/HDAC inhibition is an effective strategy for the treatment of psoriasis.

However, despite the potential success of using HDAC inhibitors, which are amides and hydroxamic acids, as anti-inflammatory therapy for psoriasis, there are limitations and gaps in the current state of science. First, the number of publications devoted to HDAC inhibitors for the treatment of psoriasis is an order of magnitude smaller than for the treatment of cancer and neurodegenerative diseases. Second, studies are needed not only on immortalized human keratinocytes but also on cellular monocytes/macrophages.

## 10. Conclusions

Thus, the theoretical and practical results obtained in the reviewed works allow us to consider histone deacetylase inhibitors as promising drug candidates for further preclinical and clinical development.

Histone deacetylase inhibitors belonging to the class of hydroxamic acids demonstrate excellent antitumor activity and neuroprotective, cardioprotective, and anti-inflammatory action. The inclusion of histone deacetylase inhibitors in the therapeutic arsenal will expand effective treatment options for cancer and diseases of the nervous, cardiovascular, respiratory, and digestive systems. Integration of histone deacetylase inhibitors with monoclonal antibodies, targeted drugs, and immunotherapy will lead to increased efficiency and patient outcomes. Combination therapy with hydroxamic acids in combination with commercially available drugs will increase the survival of patients with Alzheimer’s disease, Parkinson’s disease, pulmonary fibrosis, and psoriasis, and therapy with hydrazides will improve the quality of life of patients with liver steatosis.

However, to predict diseases at early stages, it is necessary to identify biochemical markers in the blood. For a deeper understanding of the pathogenesis of diseases and identification of common pathological biochemical cascades at the stages of early pathogenesis, it is necessary to study the mechanisms of action of histone deacetylase inhibitors at the molecular level, as well as the translational control of gene expression. Further, of great importance for the rational search for new drugs is the construction of an integrated system of biological testing.

In addition, as a promising direction for our future research, which was not included in this review, it is worth noting studies involving PROTAC-HDAC, which contain an additional linker and a ligand that recruits ubiquitin ligase and are superior to traditional HDAC inhibitors in many respects. In particular, they promote the selective degradation of HDAC through the ubiquitin–proteasome system, have stronger antiproliferative activity, and require lower therapeutic concentrations while maintaining a prolonged effect [132,133,134].

## Figures and Tables

**Table 1:** Chemical structure and inhibitory activity of HDAC inhibitors.

Chemical Structure	IC_50_ HDAC6 (nM)	IC_50_ HDAC1 (nM)
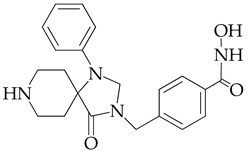	23.3	1230
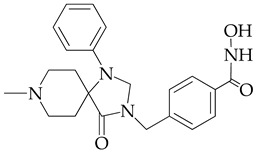	13.3	832
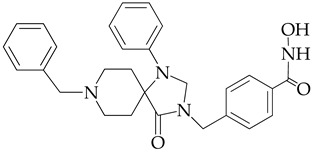	12.9	1370
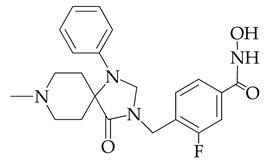	7.7	2118
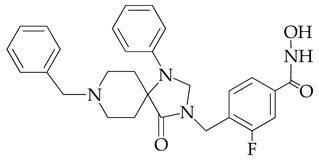	17.6	7373.2
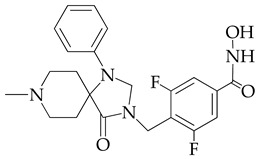	10.4	6101.3
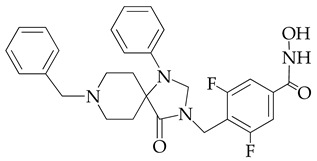	9.5	7374.5

**Table 2:** Structure of the HDAC inhibitor and the results obtained in the work.

Structure	Cancer	Results	Reference
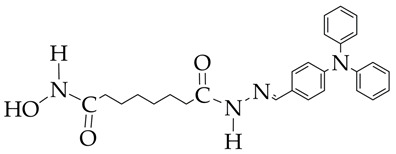 WT161 is at the preliminary preclinical studies stage.	Melanoma	1. Increased expression of Lys40-acetylated α-tubulin, confirming selective inhibition of HDAC6. 2. Cytotoxicity towards melanoma cell lines CHL-1 (IC_50_ 5.94 μM), SK-MEL-147 (IC_50_ 11.75 μM), and WM1366 (IC_50_ 26.38 μM), after 48 h of observation in the MTT test. 3. Disruption of the growth of multicellular tumor spheroids of melanoma cells, with a decrease in their size by the end of the ninth day. 4. A decrease in the clonogenic capacity of melanoma cells. 5. Enhancement of chemo-induced apoptosis with a combination of 180 μM temozolomide and 2 μM HDAC inhibitor. 6. Inhibition of the migratory capacity of melanoma cells at 6 μM.	[75]
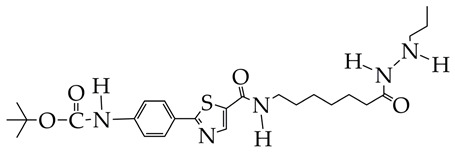		1. Inhibitory activity against HDAC3 (IC_50_ 311 nM), with a selectivity index against HDAC6 and HDAC8 greater than 32. 2. Antiproliferative activity against mouse skin melanoma B16-F10 cells (IC_50_ 5.22 μM). 3. Inhibition of migration and invasion of B16-F10 cells at a concentration of 5 μM (inhibition degree 70%). 4. Induction of tumor cell apoptosis (46% at 5 μM). 5. Induction of G2/M phase arrest (regulator of DNA replication in tumor cells). 6. Increased Ac-H3 expression according to Western blotting data. 7. Low oral exposure and low oral bioavailability in an in vivo study in Sprague–Dawley (SD) rats. 8. Inhibition in melanoma growth at a dose of 50 mg/kg (in vivo study with inoculation of B16–F10 cells into the inguinal region of mice).	[76]
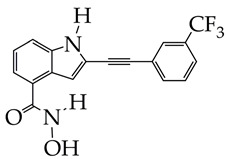	Acute myeloid leukemia	1. Significant inhibitory activity against the most active isoform of HDAC11 (IC_50_ 47 nM). 2. Suppression of growth of U937 and OCI-AML2 cell lines, with an IC_50_ value of about 10 μM. 3. Ferroptosis-inducing ability of the inhibitor to increase the level of lipid peroxidation and ROS was established. 4. Synergistic antitumor effects were established in combination with daunorubicin and doxorubicin. 5. Acceptable metabolic stability and bioavailability when administered intraperitoneally in vivo to ICR mice. 6. More powerful antitumor effect in vivo in combination with cytarabine compared to monotherapy.	[77]
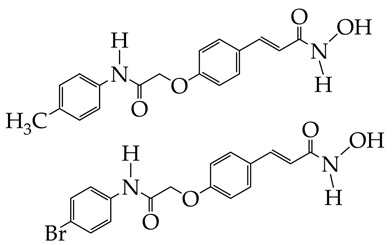	Leukemia	1. Inhibition of proliferation of human leukemia monocytic Tohoku Hospital Pediatrics-1 (THP-1) cell line (IC_50_ 1.6–2.2 μM at 48 h). 2. Inhibition of class I and II HDAC activity and increase in acetylation of histones H2a, H2b, H3, and H4.	[78]
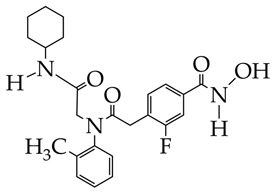		1. HDAC6 inhibition (IC_50_ 9 nM), with a selectivity coefficient relative to HDAC1 of 25. 2. Antiproliferative activity against the three leukemia cell lines HAL01 (B-cell acute lymphoblastic leukemia or B-ALL), HL60 (acute myeloid leukemia or AML), and Jurkat (T-cell acute lymphoblastic leukemia or T-ALL). IC_50_ values were 2.04–3.08 μM. 3. No cytotoxic effect on normal fibroblasts F107 and F188. 4. Synergistic effect of combination with decitabine (ZIP synergy index about 60) against AML cell lines.	[79]
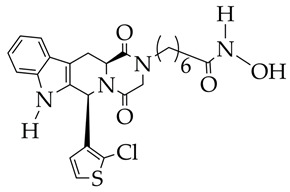	Colorectal cancer	1. Inhibitory activity against HDAC6 (IC_50_ 1.3 nM) with selectivity index against HDAC1 1009. 2. Antiproliferative effect against COLO 205, HCC-2998, HCT-116, HT29, KM12, and SW-620 cells (IC_50_ 4.3–6.47 μM). 3. Minimal cytotoxic effects against non-cancerous HaCaT (human keratinocytes) and 3 T3-L1 (mouse embryo fibroblasts) cells with viability of 87.8–88.3%. 4. Induction of apoptosis and suppression of migration in HCT-116 cells. 5. Reduction in phosphorylated ERK1/2 levels according to Western blotting.	[80]
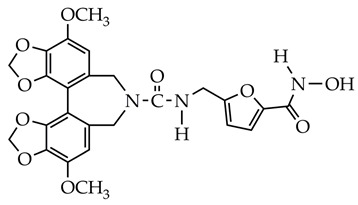 TDH-11 is at the preliminary preclinical studies stage.		1. Inhibits tumor behavior of HCT116, SW480, HT-29, and RKO cells (IC_50_ 100, 115, 169, 201 nM, respectively) and promotes apoptosis and cell cycle arrest. 2. Low cytotoxicity to normal colon epithelial cells NCM460. 3. Inhibition of HDAC1 and HDAC8 activity. 4. Inhibition of migration and invasion of HCT116 and SW480 cells. 5. Slowing down tumor growth in vivo in a mouse xenograft model (HCT-116 cells). 6. Reduction in the number of pulmonary metastatic nodes compared to the control group (no treatment).	[81]
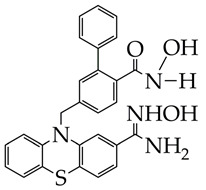	Gastric cancer	1. Inhibition of HDAC9 activity (IC50 40 nM). 2. Antiproliferative effect against SC-M1 (IC_50_ 5.8 μM), induction of apoptosis, and DNA damage.	[82]

**Table 4:** Chemical structures of HDAC inhibitors for Parkinson’s disease treatment.

HDAC Inhibitors	Reference
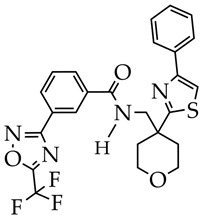 TMP269 is at the preliminary preclinical studies stage.	[98]
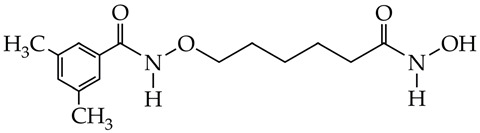 LMK-235 is at the preliminary preclinical studies stage.	[99]
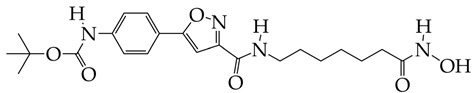 CAY is at the preliminary preclinical studies stage.	[100]

**Table 5:** Chemical structures of HDAC inhibitors and cardiovascular pathology.

HDAC Inhibitors	Pathology	Reference
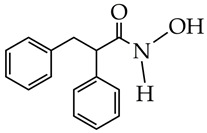 YAK577 is at the preliminary preclinical studies stage.	Heart failure	[107]
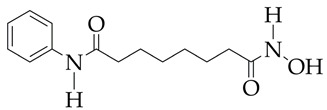 SAHA, vorinostat (is at the preliminary preclinical studies stage for cardiovascular diseases).	Dilated cardiomyopathy	[108]
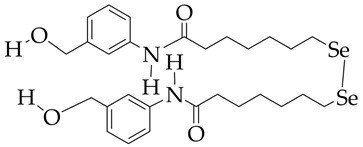 Se-SAHA is at the preliminary preclinical studies stage.	Heart failure, cardiomyopathy	[110]
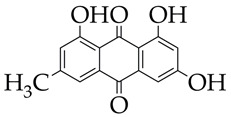 Emodin is at the preliminary preclinical studies stage.	Pathological cardiac hypertrophy	[111]

**Table 6:** Chemical structures of HDAC11 inhibitors.

Chemical Structure	IC_50_ HDAC11, nM	Reference
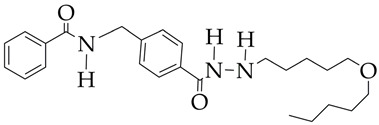	51.1	[112]
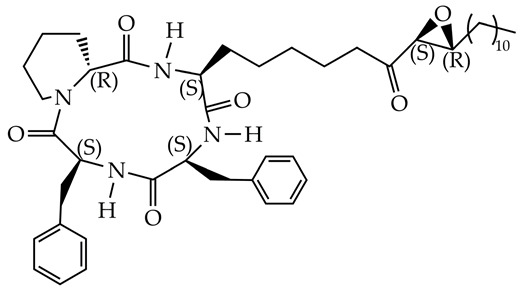 TD034	5.1	[115]
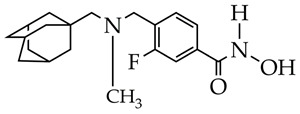 PB94	108	[116]

**Table 7:** Chemical structures of HDAC11 inhibitors and results.

Structure	Results	Reference
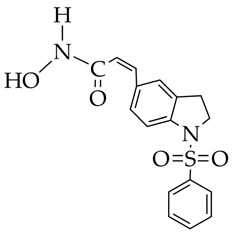 MPTOE028	1. Suppression of CTGF expression in human lung fibroblasts (WI-38 cell line). 2. Inhibition of JNK, p38, and ERK phosphorylation. 3. Reduction in α-SMA, fibronectin, and collagen production. 4. Attenuation of bleomycin-induced pulmonary fibrosis in C57BL/6 mice.	[121]
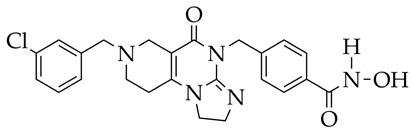	1. Inhibition of HDAC6 activity (94.8% at 1 μM, IC_50_ 42.9 nM). 2. Increase in Ac-α-tubulin and Ac-H4 levels. 3. Slowing down of weight loss in a mouse model of pulmonary fibrosis.	[122]
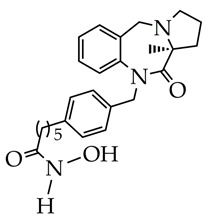	1. Inhibition of HDAC6 activity induced by TGF-β1 in HELF cells (IC_50_ 8.45 nM). 2. Stability in plasma with a half-life of 289 min and in human liver microsome (145 min). 3. Inhibition of HDAC6 activity in lung tissue induced by pulmonary fibrosis. 4. Decreased collagen fiber deposition compared to the control group according to histology. 5. Dose-dependent inhibition of α-SMA and p-Smad2/3 expression in mice according to immunohistochemistry.	[123]
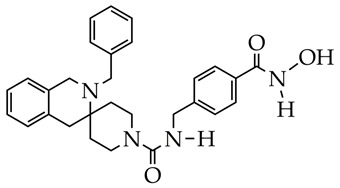	1. Selective inhibition of HDAC6 (IC_50_ 63 nM) compared to HDAC1, HDAC3, HDAC5, HDAC8, HDAC10, and HDAC11. 2. Decreased fibronectin and collagen expression in an ex vivo IPF model.	[124]

**Table 8:** Chemical structures of HDAC11 inhibitors and studies.

Chemical Structure	Studies	Reference
RGFP966 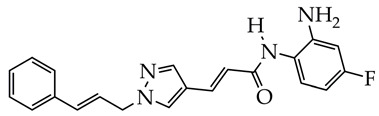	Psoriasis-like inflammatory cell model established, intracellular ROS measurement, mitochondrial membrane potential assay, immunofluorescence staining, histological analysis, enzyme-linked immunosorbent assay, and Western blot analysis	[130]
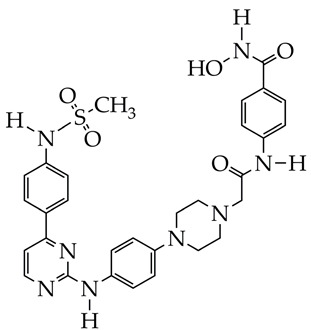	In vitro cell viability assay, NO determination assay, Western blot analysis, imiquimod-induced psoriasis mice model	[131]

## Data Availability

The data presented in this study are available on request from the corresponding author.

## Abbreviations

The following abbreviations are used in this manuscript:

ATP	Adenosine triphosphate
Bcl-2	B-cell lymphoma 2
DMSO	Dimethylsulfoxide
E2F1	Transcription factor, a protein encoded in humans by the E2F1 gene
FDA	Food and Drug Administration
HDAC	Histone deacetylase
IPF	Idiopathic pulmonary fibrosis
MASLD	Metabolic dysfunction-associated steatotic liver disease
Mcl-1	Myeloid cell leukemia 1
NAD	Nicotinamide adenine dinucleotide
NF-κB	Nuclear factor κB
p53	Tumor suppressor protein
PAK1	P21 activated kinase 1
PI3K	Phosphoinositide 3-kinase
RPD3	Proteins involved in regulating gene expression in response to heme levels
PROTAC	Proteolysis targeting chimera
SAHA	Suberoylanilide hydroxamic acid
STAT3	Signal transducer and activator of transcription 3
TNBC	Triple negative breast cancer
XIAP	X-linked inhibitor of apoptosis
ZBG	Zinc binding group
ZMF-25	Pyrido[2,3-d] pyrimidin-7(8*H*)-one-coupled hydroxamic acid
JAK	Janus kinases
